# The composition and function of the gut microbiota of Francois’ langurs (*Trachypithecus francoisi*) depend on the environment and diet

**DOI:** 10.3389/fmicb.2023.1269492

**Published:** 2023-11-16

**Authors:** Yue Sun, Yanze Yu, Ankang Wu, Chao Zhang, Xun Liu, Changjiang Qian, Jianfeng Li, Jingcheng Ran

**Affiliations:** ^1^School of Biological Sciences, Guizhou Education University, Guiyang, China; ^2^Guizhou Fanjingshan Observation and Research Station for Forest Ecosystem, Tongren, China; ^3^Guizhou Caohai Observation and Research Station for Wet Ecosystem, Bijie, China; ^4^Wildlife Institute of Heilongjiang Province, Harbin, China; ^5^Mayanghe National Nature Reserve Administration, Tongren, China; ^6^Guizhou Forest Wildlife Park, Guiyang, China; ^7^Key Laboratory of Biological Resources Exploitation and Utilization in Colleges and Universities of Guizhou Province, Guizhou Education University, Guiyang, China; ^8^Guizhou Academy of Forestry Sciences, Guiyang, China

**Keywords:** François’ langur, diet flexibility, dietary, gut microbiota, captive environment

## Abstract

The microbiota is essential for the extraction of energy and nutrition from plant-based diets and may have facilitated primate adaptation to new dietary niches in response to rapid environmental shifts. In this study, metagenomic sequencing technology was used to analyze the compositional structure and functional differences of the gut microbial community of Francois’ langurs (*Trachypithecus francoisi*) under different environmental and dietary conditions. The results showed that in terms of the composition of the gut microbial community, there were significant differences among the gut microbiota of Francois’ langurs (anthropogenic disturbed populations, wild populations, and captive populations) under different environmental and dietary conditions. The microbial communities with the highest abundance in Francois’ langurs were Firmicutes and Bacteroidetes. Firmicutes was the most abundant phylum in anthropogenic disturbed Francois’ langurs and the least abundant in captive Francois’ langurs. The abundance of Bacteroidetes was highest in captive Francois’ langurs. In the analysis and comparison of alpha diversity, the diversity of the gut microbiota of Francois’ langurs affected by anthropogenic disturbance was the highest. The significant differences in gut microbiota between Francois’ langurs in different environments and different diets were further supported by principal coordinate analysis (PCoA), with the disturbance group having a gut microbiota more similar to the wild group. Kyoto Encyclopedia of Genes and Genomes (KEGG) functional annotation analysis indicated a high abundance of functional genes involved in carbohydrate metabolism, amino acid metabolism, replication and repair, cofactor and vitamin metabolism, and other amino acid metabolism pathways. Additionally, the functional genes involved in carbohydrate metabolism pathways were significantly enriched in the gut microbial community of Francois’ langurs that were anthropogenic disturbed and captive. The gut microbiota of the Francois’ langurs exhibited potential plasticity for dietary flexibility, and long-term food availability in captive populations leads to changes in gut microbiota composition and function. This study explored the composition and function of the gut microbiota of Francois’ langurs and provided a scientific basis for understanding the physiological and health status of Francois’ langurs, effectively protecting the population of wild Francois’ langurs and reintroducing captive Francois’ langurs into the wild.

## 1 Introduction

In recent years, explosion in metagenomic techniques has enabled us to explore the complicated microbiota inhabiting micro-environments within the gastrointestinal tract (Handelsman et al., 1998; Handelsman, 2004; Muegge et al., 2011). Evidence has been accumulated to show that the gut microbiota is critical in disease, nutrition, immune responses, and development of the host (Guarner and Malagelada, 2003; Versalovic and Relman, 2006). Because the gut microbial community is highly flexible, it, in turn, affects the host’s ability to respond quickly to environmental changes (David et al., 2014; Suzuki and Ley, 2020). Multiple studies have shown that habitat-caused changes in diet and surroundings have a marked effect on the gut microbiota (Clayton et al., 2018; Amato et al., 2019; Baj et al., 2019; West et al., 2019). Additionally, food is one of the main factors affecting the gut microbiota in animals (Clayton et al., 2016). Food not only causes changes in animal gut microbial composition, metabolites, and short-chain fatty acids but also affects animal gut microbial metabolites and chemical reactions in the gut. Reduced or sustained intake of macronutrients such as fiber may lead to the loss of key microbial taxa (Wu et al., 2011; David et al., 2014; Uhr et al., 2019). Leaf-eating primates generally have longer digestive tracts to increase food residence time and improve the breakdown of fiber and secondary metabolites, and gut microbiota tend to be enriched in pathways associated with amino acid production. In contrast, the gut microbiota of non-leaf-eating primates is rich in microbe associated with starch and monosaccharide degradation. Compared with wild populations, captive populations have lower intake of crude fiber (15%) and protein (13%) and higher intake of non-structural carbohydrates (60%) and fat (12%) (Chen S. T. et al., 2018; Guo et al., 2018). A previous study demonstrated significant differences in the gut microbiota of captive and wild Guizhou snub-nosed monkeys (Hale et al., 2019). Therefore, we hypothesize that there are significant differences in the composition, structure, and function of the gut microbial community in Francois’ langurs under different environmental conditions and that these differences are related to changes in diet and environment.

Francois’ langurs (*Trachypithecus francoisi*) belong to the family Cercopithecidae, the subfamily Colobinae, and the genus *Trachypithecus*. They are mainly distributed in Chongqing, Guizhou, and Guangxi, China and the karst stone mountain area in northern Vietnam, with a population of 1,600–1,900 individuals. Francois’ langurs are national first-class protected wild animals in China and are listed as an endangered (EN) species by the International Union for Conservation of Nature (IUCN) (Niu et al., 2019). The group of Francois’ langurs in the Mayanghe National Nature Reserve Administration in Guizhou Province not only frequents the villages where humans live. The pure wild Francois’ langurs population is mainly leaf-eating. For the anthropogenic disturbed wild population, in addition to the main leafy plants, other high-sugar and high-salt protein and carbohydrate foods are common. The captive populations mainly rely on fruits and leaves as their main food. At present, the distribution and differences in microbiota between different dietary populations are still unclear, and the functions of the gut microbiota in the adaptation process to different diets need to be further studied.

In this study, to elucidate how the gut microbial community of Francois’ langurs responds to different environments and foods, metagenomic sequencing was performed to compare three different dietary patterns: the composition and function of the gut microbiota in the anthropogenic disturbed group (AD), wild group (W), and captive group (C) Francois’ langurs. The research objectives included (1) determining the differences in the composition and diversity of the gut microbiota of Francois’ langurs under different environmental and dietary conditions and (2) determining the adaptability of the gut microbiota in Francois’ langurs to the environment under different environmental and dietary conditions and the significantly different functional characteristics of the gut microbial community. This study further served to evaluate the health status of wild Francois’ langur populations under different environmental conditions, provide a scientific basis for the protection of Francois’ langurs in the Mayanghe National Nature Reserve Administration, and provide a theoretical basis for the breeding of captive Francois’ langurs.

## 2 Materials and methods

### 2.1 Study site and sample collection

The research sites were the Mayanghe National Nature Reserve Administration in Guizhou Province and the Guizhou Forest Wildlife Zoo in Guizhou Province. The Mayanghe National Nature Reserve Administration is located at the junction of Yanhe Tujia Autonomous County and Wuchuan Gelao and Miao Autonomous County in Guizhou Province, with a total area of 311.13 km^2^ (Niu et al., 2019). The landform types are mainly valleys and karst formations, and the annual average temperature is 16.7°C. The Francois’ langurs population in the Mayanghe National Nature Reserve Administration in Guizhou Province includes a pure wild population (wild group, *n* = 5) and an anthropogenic disturbed population (anthropogenic disturbed group, *n* = 4). The anthropogenic disturbed population, which is artificially disturbed by humans, lives near the protection station of the Mayanghe National Nature Reserve Administration and often moves along highways. Anthropogenic disturbed monkeys are likely to ingest anthropogenic foods (fruits, peanuts, etc.). The Francois’ langurs population at the Guizhou Forest Wildlife Zoo is a captive population (captive group, *n* = 4). From May to July 2022, 17 transects were set up within the potential habitat range of the wild population of Francois’ langurs. During the 120 observation days, the instant scanning method was used to track 5–7 wild population groups. Fecal samples were collected using sterile disposable gloves immediately after Francois’ langur defecated. The gloves were changed after each sample was collected, and the fecal samples were immediately stored at −80°C. The collected feces of the wild Francois’ langurs population was from various social groups with one male and multiple females. The feces of four different individuals of captive Francois’ langurs were collected at Guizhou Forest Wildlife Zoo. Because the Francois’ langurs were kept in different cages at night, feces were collected every morning immediately after defecation. All fecal samples were stored at −80°C immediately after collection until laboratory analysis.

### 2.2 Dietary composition of captive Francois’ langurs

The research site for captive Francois’ langurs was the Francois’ langurs cage in the primate exhibit at the Guizhou Forest Wildlife Zoo, located in Zhazuo Town, Xiuwen County, Guizhou Province. The research subjects were 4 Francois’ langurs, i.e., 2 adult males and 2 adult females. The four individuals were not related to each other. Their diet consisted mainly of steamed cornbread, eggs, fruits and vegetables, and wild plants in small amounts.

### 2.3 Extraction and testing of samples

Total microbial genomic DNA samples were extracted from feces using the OMEGA Mag Bind Soil DNA Kit (M5635-02) (OMEGA Bio-Tek, Norcross, GA, USA); the concentration, integrity, and purity of DNA were assessed using Agilent 5,400.

### 2.4 Library construction

Libraries were constructed using the NEB Next® Ultra™ DNA Library Prep Kit for the Illumina platform (NEB, USA). DNA samples were randomly cleaved into approximately 350-bp fragments using a Covaris ultrasonic crusher. DNA fragments underwent end-repair, the addition of poly-A tails, the addition of sequencing connectors, purification, and PCR amplification to prepare the libraries. The PCR products were purified using an AMPure XP system; the insert size of the library was assessed using an Agilent 2,100, and the library concentration was quantified using real-time PCR. DNA libraries were sequenced using the Novaseq6000 high-throughput sequencing platform.

### 2.5 Bioinformatics analysis

#### 2.5.1 Data quality control and de-host sequences

The Illumina NovaSeq high-throughput sequencing platform was used for metagenomic sequencing to obtain raw metagenomic data of bacteria, fungi, and viruses in the fecal samples from Francois’ langurs. To ensure the reliability of the data, KneadData software was used to preprocess the raw sequencing data. The specific processing steps were as follows: (1) The adapter sequences in the raw data (based on Trimmomatic, parameter: ILLUMINACLIP: adapters_path:2:30:10), low-quality (default quality score threshold ≤20) sequences (based on Trimmomatic, parameter: SLIDINGWINDOW:4:20), and sequences with a final length less than 50 bp (based on Trimmomatic, parameter: MINLEN:50) were removed. (2) Considering that there may be host contamination in the specimens, the clean data were aligned to the host genome. Bowtie2 software was used^1^ to filter sequences from the host to obtain valid sequences for subsequent analysis. (3) Finally, the rationality and effect of quality control were tested using FastQC (Mckenna et al., 2010; Martin, 2011; Schmieder and Edwards, 2011; Langmead and Salzberg, 2012).

#### 2.5.2 Species annotation

Alignment with Kraken2 and the self-built microbial nucleic acid database (screening sequences belonging to bacteria, fungi, archaea, and viruses in the NCBI NT nucleic acid database and RefSeq genome-wide database) was used to calculate the number of sequences of species in the samples, and then, Bracken was used to predict the actual relative abundance of species in the samples. Kraken2 is a recently developed alignment software based on K-mer. The local Kraken2 database contains 16,799 known bacterial genomes (Wood and Salzberg, 2014; Brum et al., 2015; Mandal et al., 2015; Lu et al., 2017).

#### 2.5.3 Functional annotation based on reads

Using HUMAnN2 software, the quality controlled and de-host sequences were aligned with the protein database (UniRef90) (based on DIAMOND), and annotation information and a relative abundance table for each functional database were obtained based on the correspondence between the UniRef90 ID and each database (Segata et al., 2011; Zhu et al., 2012; Kim et al., 2016; Franzosa et al., 2018). Using the species abundance table and functional abundance table, abundance clustering analysis, principal coordinates analysis (PCoA), NMDS dimensionality reduction analysis (species only), and sample clustering analysis were performed; using the grouping information, LEfSe biomarker analysis and the Dunn test were performed to evaluate differences in species composition and functional composition between samples (Villar et al., 2015).

## 3 Results

### 3.1 Gut microbiota composition of Francois’ langurs

**Figure 1:**
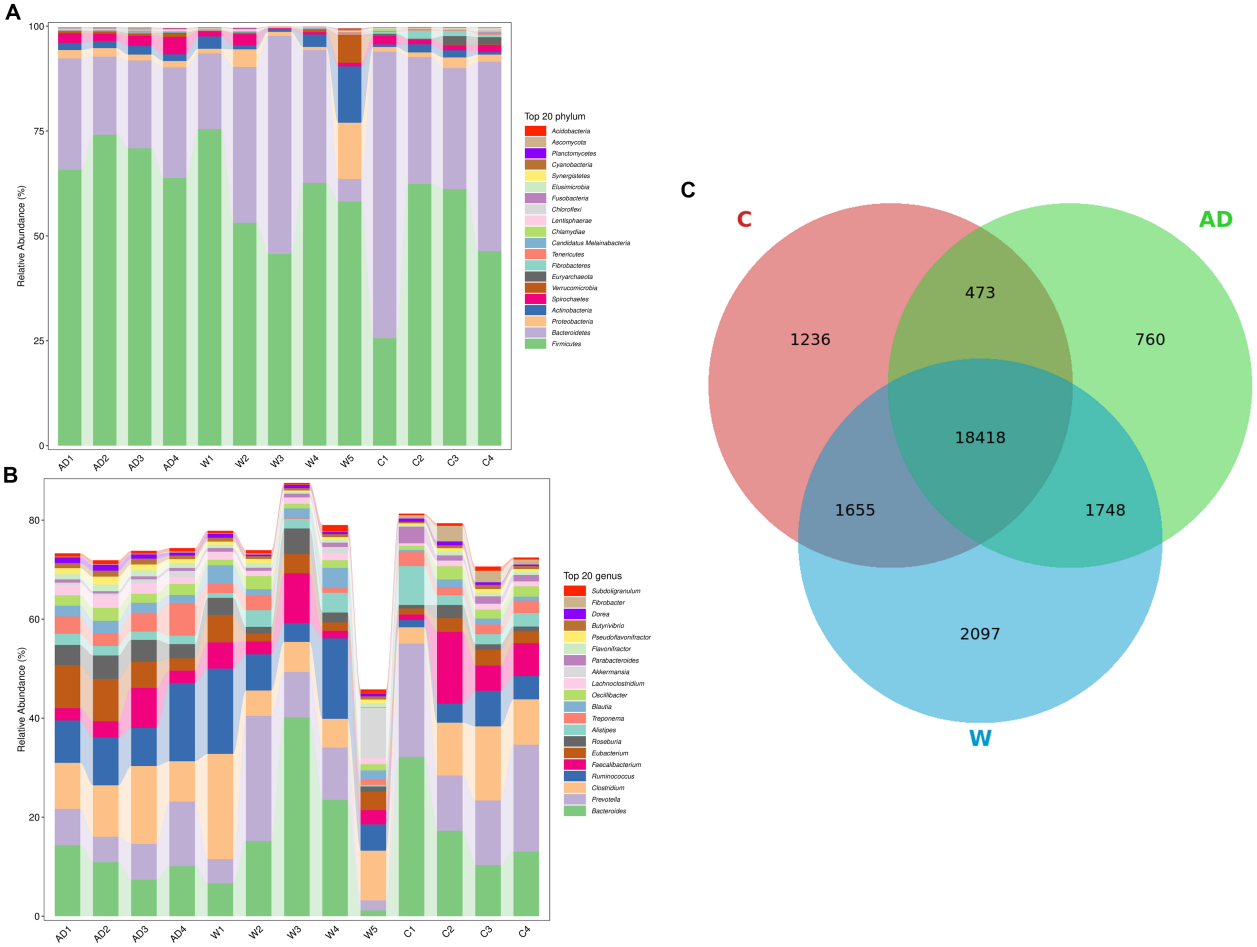
**(A)** Histogram of the relative abundance at the phylum level. Relative abundance (%) of the 20 most abundant bacterial phyla and genera obtained from 13 fecal samples of Francois’ langurs. **(B)** Histogram of the relative abundance at the genus level. Relative abundance (%) of the 20 most abundant bacterial phyla and genera obtained from 13 fecal samples of Francois’ langurs. **(C)** Species Venn diagram obtained from 13 fecal samples of Francois’ langurs. Each ellipse represents a group, the overlapping area between the ellipses indicates the common species among the groups, and the non-overlapping area indicates the unique species of the corresponding sample; the number in each block indicates the number of common or unique species in the groups contained in the block. AD, anthropogenic disturbed populations; W, wild populations; C, captive populations.

A total of 90,199,172.27 Mbp raw data were obtained from 13 Francois’ langurs feces samples. After quality control, 89,021,440.65 Mbp valid data were obtained, corresponding to 603,864,550 reads, and all samples passed stringent quality control. The sequencing data statistics are shown in Supplementary Table S1. The percentage of bases with quality values of ≥20 or ≥ 30 reached more than 97 and 93%, respectively, indicating that the sequencing data showed high reliability. A total of 13 rarefaction curves approached a plateau, suggesting that the number of samples and sequencing depth were sufficient for experimental analyses (Supplementary Figure S1). The significant differences in the gut microbial composition of feces samples of Francois’ langurs between the anthropogenic disturbance group, wild group, and captive group. At the phylum level (Figure 1A), Firmicutes (A:0.69 ± 0.05; W:0.59 ± 0.11;C:0.49 ± 0.17) and Bacteroidetes (A:0.23 ± 0.04; W: 0.29 ± 0.18;C:0.43 ± 0.18) were the main dominant phyla in the wild group, anthropogenic interference group, and captive group, followed by Proteobacteria (A: 0.02 ± 0; W: 0.04 ± 0.05;C:0.02 ± 0.01), Actinobacteria (A:0.02 ± 0;W:0.04 ± 0.05;C:0.01 ± 0.01), and Spirochaetes (A:0.03 ± 0.01; W: 0.01 ± 0.01;C:0.02 ± 0). At the genus level (Figure 1B), Ruminococcaceae (A: 0.2 ± 0.03; W: 0.18 ± 0.05;C:0.15 ± 0.07) was the main dominant genus, followed by Bacteroides (A:0.09 ± 0.02;W:0.14 ± 0.12;C:0.15 ± 0.08), Lachnospiraceae (A:0.16 ± 0.03%;W:0.12 ± 0.03;C:0.07 ± 0.03), Prevotellaceae (A:0.08 ± 0.04;W:0.1 ± 0.08;C:0.16 ± 0.04), and Clostridiaceae (A:0.11 ± 0.03;W:0.09 ± 0.06;C:0.09 ± 0.04). According to the composition spectrum of each sample at the species level, the number of common and unique species was calculated. The number of species common and unique in the three groups was visually presented by the Venn diagram (Figure 1C).

**Figure 2:**
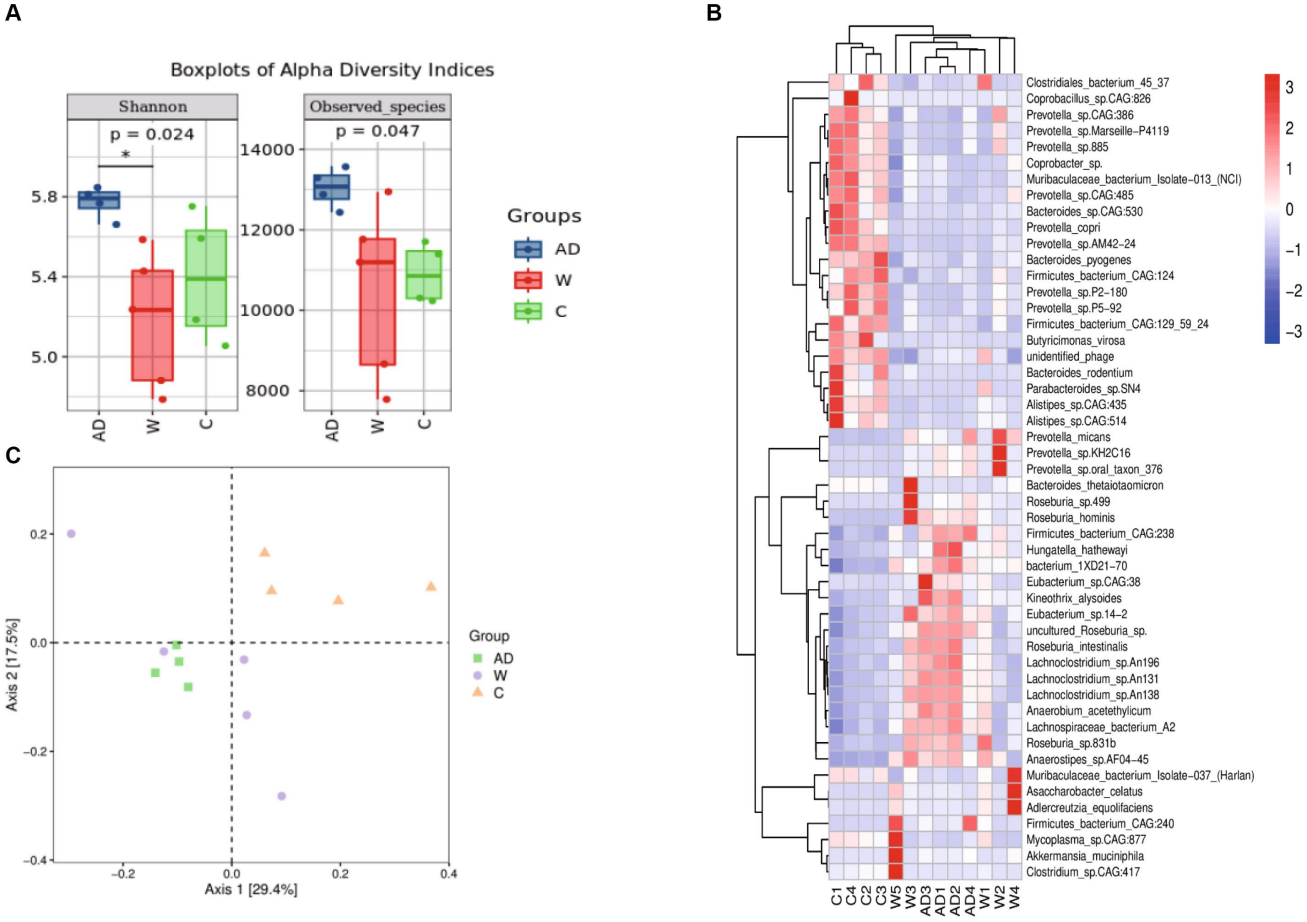
**(A)** Boxplots of alpha diversity indices showing differences between the three groups. * represents significant *p* (FDR)-value <0.05. **(B)** Principal Coordinate Analysis (PCoA) plot was built using unweighted UniFrac distances to assess beta diversity. **(C)** Species composition heatmap. The samples were first clustered based on the similarity of species composition abundance distribution between each other and then arranged horizontally based on the clustering results. Similarly, each taxonomic unit was also clustered based on the similarity of the distribution in different samples and was arranged vertically based on the clustering results. In the figure, red represents the species with higher abundance in the corresponding sample, and blue represents the species with lower abundance. AD, anthropogenic disturbed populations; W, wild populations; C, captive populations.

We further calculated significant differences in community richness based on the abundance (Figure 2A), as estimated by Shannon and observed_species indices. The Shannon index of the AD group was significantly higher than that of the W and C groups. Normally captive lifestyles or built environments will lead to a decrease in gut microbiota diversity in mammals. However, the Shannon index of the C group was significantly higher than that of the W group. However, animals that rely on a single source of food (or few sources) usually present a low diverse microbiota. Here, we found that providing a foreign, more diverse food supply may have led to a more highly diverse gut microbiota. The PCoA ordination and PERMANOVA, using unweighted Unifrac distance, further supported the significant dissimilarity in the gut microbiota community between the W and C groups (Figure 2B; PERMANOVA test: *p* < 0.01). In addition, the microbial community was more similar to the PCoA plot in the AD group compared with the W group. In this study, there is a profound difference in the gut microbiota composition between the W and AD groups within the same natural region. The top 50 relative abundant taxa with significant differences among the three groups were analyzed by clustering, and heat maps were drawn (Figure 2C).

### 3.2 Functional differences in the gut microbiota of Francois’ langur populations in different environments

**Figure 3:**
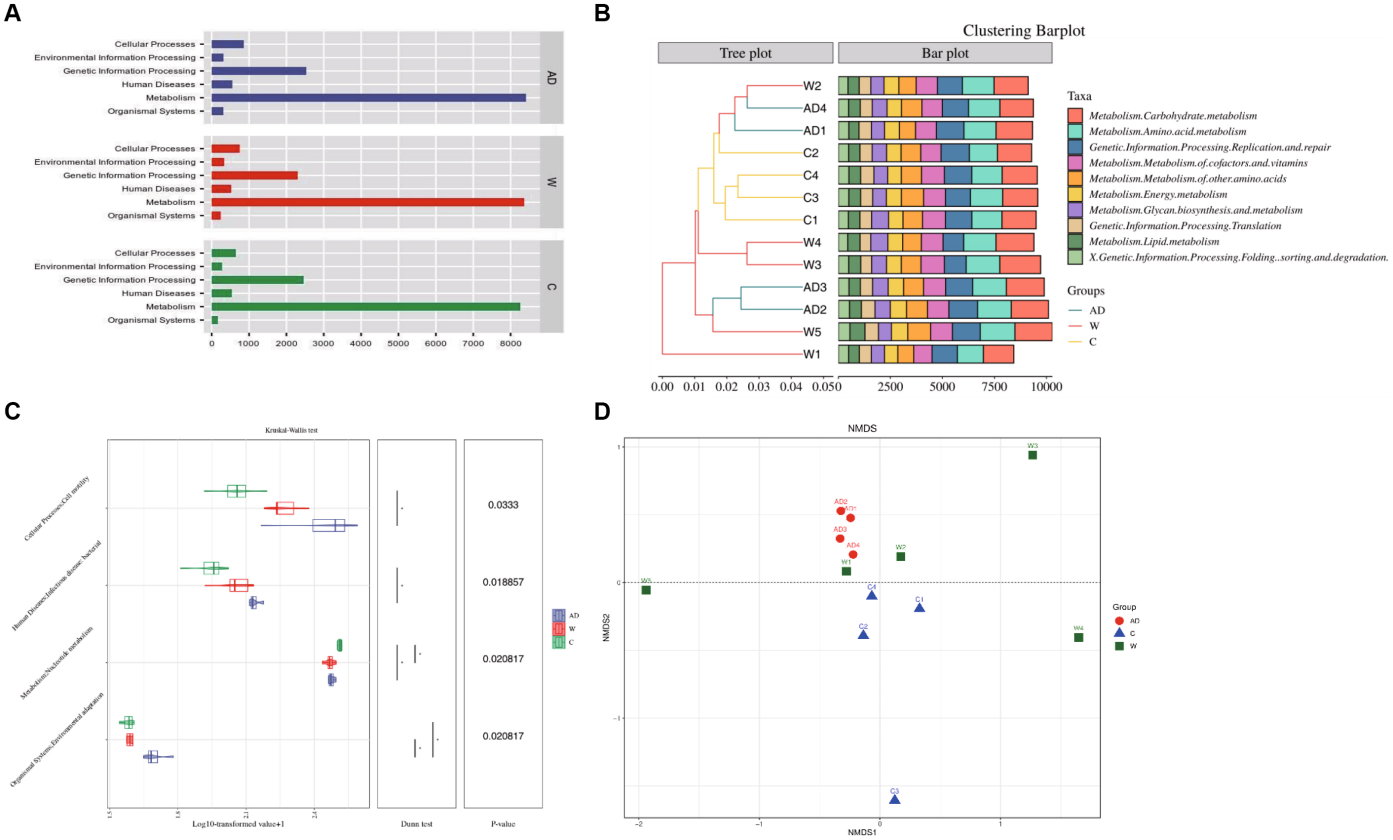
**(A)** Kyoto Encyclopedia of Genes and Genomes functional category analysis of level 1. **(B)** The combination of the clustering tree and the bar chart shows the similarity between the Kyoto Encyclopedia of Genes and Genomes level 2 pathway. **(C)** Differences in the Kyoto Encyclopedia of Genes and Genomes level 2 pathway between different groups. Samples through a combination chart of violin and box line or a bar chart with an asterisk. Statistical analysis was performed with the Wilcoxon test. **(D)** NMDS plot of unweighted UniFrac distances of fecal microbiota composition of individual Francois’ langurs. AD, anthropogenic disturbed populations; W, wild populations; C, captive populations.

Metagenomic analysis confirmed 7,921 KOs and 45 KEGG Level 2 categories (Supplementary Table S2; Supplementary Figure S2). A total of 993,161 genes were annotated and mapped to the KEGG pathway, and the number of genes annotated to the carbohydrate, amino acid, and energy metabolism pathways ranked to be the top three. Functional gene annotations to primary pathways with the most abundance including Metabolism, Genetic Information Processing, Cellular Processes, Human Diseases, Organismal Systems, Environmental Information Processing (Figure 3A). Cluster bar plots show the abundance of the top 10 genes annotated to KEGG secondary pathways in each metabolic pathway. The cluster bar plots annotated to the KEGG secondary pathway showed the highest similarity among captive langur samples (Figure 3B). The number of genes were annotated to the carbohydrate metabolism, amino acid metabolism, replication and repair, metabolism of cofactors and vitamins, and metabolism of other amino acid pathways ranked to be the top five (Figure 3B). The top 6–10 metabolic pathways were energy metabolism, glycan biosynthesis and metabolism, translation of genetic information processing, and lipid metabolism, folding, and sorting, and the degradation of genetic information processing showed differences in the number and abundance of annotated genes among the three groups (Figure 3B). The genes of the KEGG secondary pathway that show inter-group differences in various metabolic pathways are environmental adaptation, nucleotide metabolism, infectious bacterial disease, and cell motility (Figure 3C). The obtained Bray–Curtis distance matrix was analyzed by NMDS using R software. Figure 3D shows the structure distribution of community samples. The results showed that the functional composition of the AD group samples had the least difference and the highest similarity.

### 3.3 Differential analysis of functional genes in the metabolic pathways of the gut microbial community of Francois’ langurs caused by environmental changes

**Figure 4:**
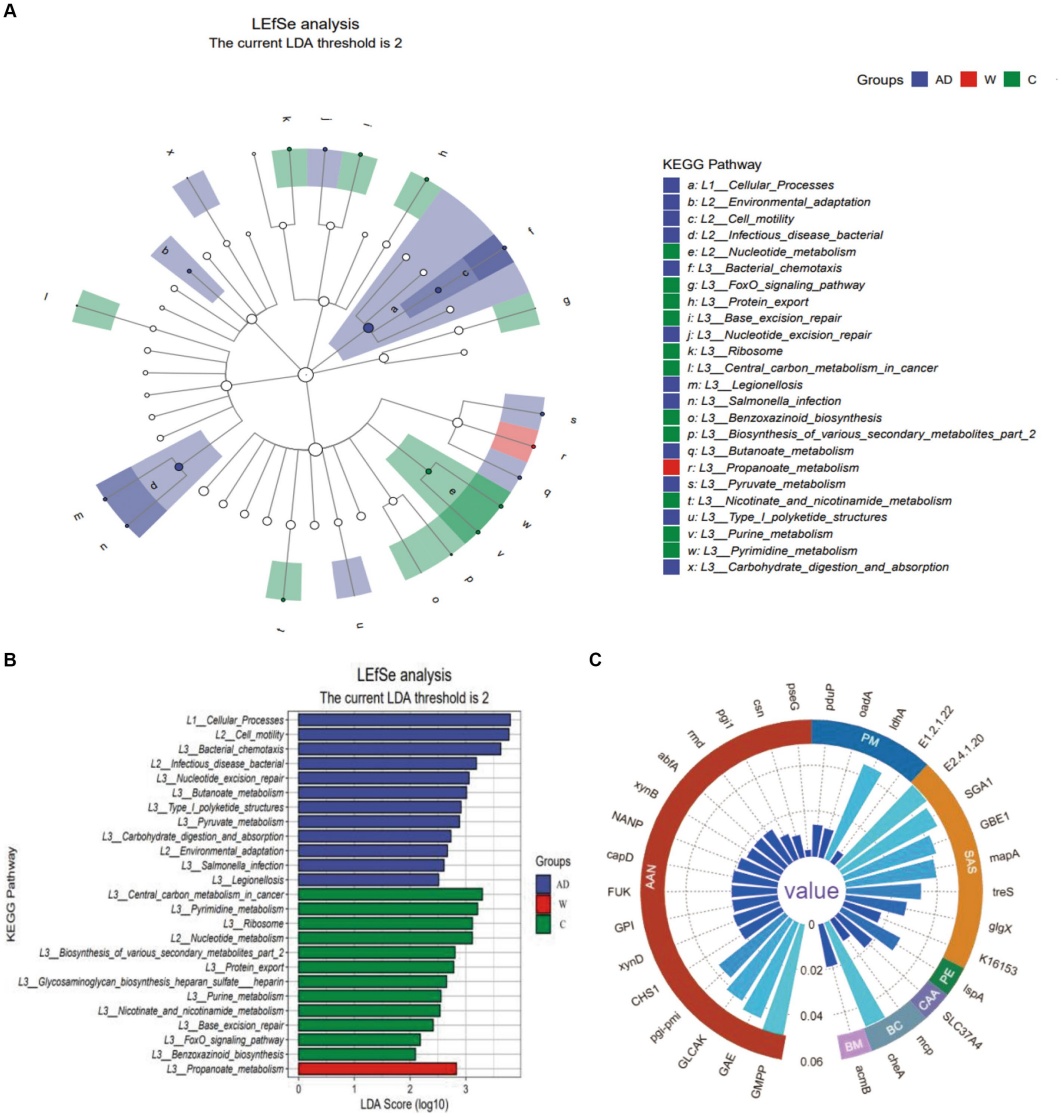
**(A)** The Kyoto Encyclopedia of Genes and Genomes level 3 pathway in LEfSe analysis (LDA > 2). **(B)** The LDA score distribution of Kyoto Encyclopedia of Genes and Genomes level 3 pathway in LEfSe analysis (LDA > 2). **(C)** The circular grouping bar chart represents genes with significant differences in the Kyoto Encyclopedia of Genes and Genomes level 3 pathway. AAN, Amino sugar and nucleotide sugar metabolism; PM, Propanoate metabolism; SAS, Starch and sucrose metabolism; PE, Protein export; CAA, Carbohydrate digestion and absorption; BC, Bacterial chemotaxis; BM, Butanoate metabolism; AD, anthropogenic disturbed populations; W, wild populations; C, captive populations.

A LEfSe functional cladogram was used to analyze the metabolic pathways with significant differences in carbohydrate metabolism, including propanoate metabolism, butanoate metabolism, pyruvate metabolism, starch and sucrose metabolism, and amino sugar and nucleotide sugar metabolism, among the three groups of Francois’ langurs (Figure 4A). Five functional genes with significant differences in carbohydrate metabolism pathways were compared and analyzed using the KW test (Figure 4B).

For the amino sugar and nucleotide sugar metabolism (ko00520) pathway, pseG (K15897), csn (K01233), and GLCAK (K16190) were only abundant in the C group; pgi-pmi (K15916), CHS1 (K00698), and (pgi-pmi) K15916 were not abundant in the C group, and NANP (K01097) was abundant only in the AD group; the remaining functional genes with significant differences are shown in the supporting data. For the starch and sucrose metabolism pathway, SGA1 (K01178) was only abundant in the AD group, and (GBE1) K00700, (mapA) K00691, treS (K05343), glgX (K02438), and K16153 were all functional genes with significant differences among the AD group, W group, and C group. For the propanoate metabolism pathway, pduP (K13922) was the functional gene with significant differences among the AD, W, and C groups, and the relative abundance of pduP (K13922) in the C group was higher than that in the AD group and the W group. For the pyruvate metabolism pathway, E1.2.1.22 (K19266) was only abundant in the C group, and oadA (K01571) and ldhA (K03778) were functional genes with significant differences among the AD group, W group, and C group. For the butanoate metabolism pathway, acmB (K18372) was only abundant in the AD group. Regarding carbohydrate digestion and absorption in the digestive system, SLC37A4 (K08171) was only abundant in the AD group.

Among the metabolic pathways of protein export in the three groups of Francois’ langurs, lspA (K03101) differed among the three groups, with the highest abundance in the C group. Among the bacterial chemotaxis metabolic pathways of cell processes in the three groups of Francois’ langurs, mcp (K03406) and cheA (K03407) were different among the three groups, and the C group had the lowest abundance (Figure 4C).

## 4 Discussion

This is the first study to reveal the differences in the structure and function of gut microbiota in Francois’ langurs under different environmental and dietary conditions through fecal metagenomic data. Dietary patterns are an important determinant of gut microbial diversity. Our findings suggest that microbiota analyses are important for langur ecology and conservation. The structure and composition, diversity, and function of gut microbes in Francois’ langurs changed with different environments and diets.

First, the predominant phyla of Francois’ langurs were Firmicutes and Bacteroidetes, which is similar to the findings of previous studies on wild rhesus macaques (Chen S. T. et al., 2018) and other wild primate species, such as gorilla (Gomez et al., 2015), *Rhinopithecus roxellana* (Su et al., 2016), Ethiopian chlorocebus monkeys (Trosvik et al., 2018), *Lemur catta*, and *Propithecus verreauxi* (Fogel, 2015). Second, we found that the captive environment significantly altered the gut microbial composition and structure of Firmicutes and Bacteroidetes. In this study, Firmicutes was the most abundant phylum in the anthropogenic disturbed group (AD) and least abundant in the captive group (C). Bacteroidetes was the most abundant phylum in the C group and least abundant in the AD group (Figures 2A,B). Similar results have been reported for other non-human primates (NHPs) (Clayton et al., 2016; Frankel et al., 2019; Campbell et al., 2020), and changes in captivity or habitat can cause primates to lose their native microbiota (Frankel et al., 2019). In this study, the abundance of Bacteroidetes was highest in captive Francois’ langurs and significantly different from that in the AD group and the W group. The explanation for this phenomenon may be the inadequate intake of crude fiber and excess intake of simple carbohydrates by captive monkeys. Several studies have confirmed that the diets of captive animals contain more carbohydrates and less crude fiber and protein than those of wild animals (Nijboer and Clauss, 2006; Guo et al., 2018; Liu et al., 2018; Chen Z. et al., 2018). Because Bacteroidetes promote the digestion and breakdown of polysaccharides and proteins (Spence et al., 2006), the efficiency of simple carbohydrate digestion in captive animals appears to be dependent on Prevotella (Bacteroidetes). The W group and the AD group were significantly enriched in Ruminococcaceae flora, and Ruminococcaceae degrade cellulose and hemicellulose, thus providing energy sources for the host (Biddle et al., 2013).

In this study, there were significant differences in metabolism pathways, cellular processes, and genetic information processing in gut microbial functions of Francois’ langurs under different environments. In addition, most of the key functions of the gut microbes in Francois’ langurs living in the Mayanghe National Nature Reserve Administration were related to short-chain fatty acid metabolism. Ruminococcus, Lachnospiraceae, and Clostridium, whose abundance was relatively increased, are involved in breaking down plant structural carbohydrates and producing short-chain fatty acids that can be utilized by the host (Chassard et al., 2010). Carbohydrate intake results in increased numbers of *Bacteroides* spp. and increased levels of short-chain fatty acids in feces (Hale et al., 2019). The high functional potential of short-chain fatty acids, such as butyrate, acetate, and lactate, may indicate compensation for energy intake through microbial fermentation. Studies have also shown that dietary supplementation with resistant starch increases the abundance of *Ruminococcus bromii* and *Bifidobacterium* and leads to increased levels of short-chain fatty acids and propionic and butyric acids in the gut (Parks et al., 2013; Belcheva et al., 2014).

In this study, there was a trend of enrichment of butyrate metabolic pathways in Francois’ langurs in the AD group, indicating that diet involves the metabolism of various cornstarch polysaccharides, which can affect multiple metabolic pathways in the host by affecting different gut flora. Different gut microbiota have different preferences for polysaccharides entering the intestine, indicating that intake of dietary polysaccharides is a strategy that can directly affect the balance of gut microbiota species (Liu et al., 2021). The results showed that tea seed meal regulated the gut microbiota of animals in the feed. The addition of 0.50% of tea seed meal could significantly reduce the content of *Escherichia coli* in the cecum of broilers. The addition of tea seed meal to the diet had a certain regulatory effect on the body fat metabolism and gut microbiota of broilers (Sun et al., 2017). In captive Francois’ langurs fed a high-protein diet, metabolic pathways for protein export were more active, suggesting an active interaction between the gut microbiota and the host. The abundance of bacterial chemotaxis genes in the gut microbiota of Francois’ langurs varies significantly with different environments, and in the wild environment, Francois’ langurs have a higher abundance of bacterial chemotaxis genes.

## 5 Conclusion

In conclusion, changes in the living environment and diet are important influencing factors for the gut microbial community. The structure, composition, diversity, and function of gut microbes in Francois’ langurs change with different environments and diets. In this study, captive Francois’ langurs had poor crude fiber digestion ability and strong simple carbohydrate digestion ability. Functional genes involved in carbohydrate metabolism pathways were significantly enriched in the gut microbial community of Francois’ langurs in the AD group and the captive group. The positive role of the gut microbiota in host dietary adaptation in captive environments and the large number of effects of captive environments on the gut microbiota suggest a complex interaction between the gut microbiota and the environment. Wild Francois’ langurs adapt to food differences through the interaction between food and gut microbial community, potentially facilitating the adjustment of their diet structure. Understanding changes in the gut microbial community of Francois’ langurs may help explain the effects of diet on animal physiology and metabolism as well as the ecological adaptation strategies of wild Francois’ langurs to habitat changes.

## Data availability statement

The data presented in this study are deposited in the NCBI database under accession number PRJNA997718.

## Ethics statement

Ethical approval was not required for the study involving animals in accordance with the local legislation and institutional requirements because this study uses strains obtained from animal feces. Guizhou Normal University did not require the study to be reviewed or approved by an ethics committee because animal feces are obtained in the wild environment or on the cement floor inside the cage, not in contact with animals and not causing any impact or harm to them.

## Author contributions

YS: Conceptualization, Data curation, Formal analysis, Investigation, Methodology, Project administration, Writing – original draft, Writing – review & editing. YY: Methodology, Software, Writing – review & editing. AW: Investigation, Resources, Supervision, Writing – review & editing. CZ: Resources, Supervision, Writing – review & editing. XL: Conceptualization, Supervision, Writing – review & editing. CQ: Data curation, Methodology, Writing – review & editing. JL: Conceptualization, Supervision, Writing – review & editing. JR: Conceptualization, Supervision, Writing – review & editing.

## Footnotes

^1^Bowtie2, http://bowtie-bio.sourceforge.net/bowtie2/index.shtml. Parameter: --very -sensitive.

## References

[ref1] Amato K. R. Sanders J. G. Song S. J. Nute M. Metcalf J. L. Thompson L. R.. (2019). Evolutionary trends in host physiology outweigh dietary niche in structuring primate gut microbiomes. ISME J. 13, 576–587. doi: 10.1038/s41396-018-0175-0, PMID: 29995839 PMC6461848

[ref01] Baj A. Moro E. Bistoletti M. Orlandi V. Crema F. Giaroni C. (2019). Glutamatergic signaling along the microbiota-gut-brain axis. Int. J. Mol. Sci. 20:1482. doi: 10.3390/ijms20061482 30934533 PMC6471396

[ref2] Belcheva A. Irrazabal T. Robertson S. J. Streutker C. Maughan H. Rubino S.. (2014). Gut microbial metabolism drives transformation of MSH2-deficient colon epithelial cells. Cells 158, 288–299. doi: 10.1016/j.cell.2014.09.041, PMID: 25036629

[ref3] Biddle A. Stewart L. Blanchard J. Leschine S. (2013). Untangling the genetic basis of fibrolytic specialization by Lachnospiraceae and Ruminococcaceae in diverse gut communities. Diversity 5, 627–640. doi: 10.3390/d5030627

[ref4] Brum J. R. Ignacio-Espinoza J. C. Roux S. Doulcier G. Acinas S. G. Alberti A.. (2015). Patterns and ecological drivers of ocean viral communities. Science 348:1261498. doi: 10.1126/science.1261498, PMID: 25999515

[ref5] Campbell T. P. Sun X. Patel V. H. Sanz C. Morgan D. Dantas G. (2020). The microbiome and resistome of chimpanzees, gorillas, and humans across host lifestyle and geography. ISME J. 14, 1584–1599. doi: 10.1038/s41396-020-0634-2, PMID: 32203121 PMC7242348

[ref6] Chassard C. Delmas E. Céline R. Bernalier-Donadille A. (2010). The cellulose-degrading microbial community of the human gut varies according to the presence or absence of methanogens. FEMS Microbiol. Ecol. 74, 205–213. doi: 10.1111/j.1574-6941.2010.00941.x 20662929

[ref7] Chen S. T. Luo X. Hou R. Raubenheimer D. Ji W. Jin X.. (2018). Nutrient balancing by captive golden snub-nosed monkeys (*Rhinopithecus roxellana*). Int. J. Primatol. 39, 1124–1138. doi: 10.1007/s10764-018-0070-6

[ref8] Chen Z. Yeoh Y. K. Hui M. Wong P. Y. Chan M. C. W. Ip M.. (2018). Diversity of macaque microbiota compared to the human counterparts. Sci Reports 8, 15573–15515. doi: 10.1038/s41598-018-33950-6, PMID: 30349024 PMC6197227

[ref9] Clayton J. B. Gomez A. Amato K. Knights D. Travis D. A. Blekhman R.. (2018). The gut microbiome of nonhuman primates: lessons in ecology and evolution. Am. J. Primatol. 80:e22867. doi: 10.1002/ajp.22867 29862519

[ref10] Clayton J. B. Vangay P. Hu H. Ward T. Hillmann B. M. Al-Ghalith G. A.. (2016). Captivity humanizes the primate microbiome. Proc. Natl. Acad. Sci. U. S. A. 113, 10376–10381. doi: 10.1073/pnas.1521835113, PMID: 27573830 PMC5027417

[ref11] David L. A. Maurice C. F. Carmody R. N. Gootenberg D. B. Button J. E. Wolfe B. E.. (2014). Diet rapidly and reproducibly alters the human gut microbiome. Nature 505, 559–563. doi: 10.1038/nature12820, PMID: 24336217 PMC3957428

[ref12] Fogel A. T. (2015). The gut microbiome of wild lemurs: a comparison of sympatric lemur catta and *Propithecus verreauxi*. Folia Primatologica. Int. J. Primatol. 86, 85–95. doi: 10.1159/000369971 26022304

[ref13] Frankel J. S. Mallott E. K. Hopper L. M. Ross S. R. Amato K. R. (2019). The effect of captivity on the primate gut microbiome varies with host dietary niche. Am. J. Primatol. 81:e23061. doi: 10.1002/ajp.23061, PMID: 31713260

[ref14] Franzosa E. A. McIver L. J. Rahnavard G. Thompson L. R. Schirmer M. Weingart G.. (2018). Species-level functional profiling of metagenomes and metatranscriptomes. Nat. Methods 15, 962–968. doi: 10.1038/s41592-018-0176-y, PMID: 30377376 PMC6235447

[ref15] Gomez A. Petrzelkova K. Yeoman C. J. Vlckova K. Mrázek J. Koppova I.. (2015). Gut microbiome composition and metabolomic profiles of wild western lowland gorillas (*Gorilla gorilla*) reflect host ecology. Mol. Ecol. 24, 2551–2565. doi: 10.1111/mec.13181, PMID: 25846719

[ref16] Guarner F. Malagelada R. (2003). Gut flora in health and disease. Lancet 361, 512–519. doi: 10.1016/S0140-6736(03)12489-0 12583961

[ref17] Guo S. T. Hou R. Garber P. A. Raubenheimer D. Righini N. Ji W. H.. (2018). Nutrient-specific compensation for seasonal cold stress in a free-ranging temperate colobine monkey. Funct. Ecol. 32, 2170–2180. doi: 10.1111/1365-2435.13134

[ref18] Hale V. L. Tan C. L. Niu K. F. Yang Y. Q. Zhang Q. K. Knight R.. (2019). Gut microbiota in wild and captive Guizhou snub-nosed monkeys, *Rhinopithecus brelichi*. Am. J. Primatol. 81:e22989. doi: 10.1002/ajp.22989 31106872

[ref19] Handelsman J. (2004). Metagenomics: application of genomics to uncultured microorganisms. Microbiol. Mol. Biol. Rev. 68, 669–685. doi: 10.1128/MMBR.68.4.669-685.2004, PMID: 15590779 PMC539003

[ref20] Handelsman J. Rondon R. Brady F. Clardy J. Goodman M. (1998). Molecular biological access to the chemistry of unknown soil microbes: a newfrontier for natural products. Chem. Biol. 5, R245–R249. doi: 10.1016/S1074-5521(98)90108-9 9818143

[ref21] Kim J. W. Kim M. S. Koh A. Y. Xie Y. Zhan X. W. (2016). FMAP: functional mapping and analysis pipeline for metagenomics and metatranscriptomics studies. BMC Bioinformat. 17:420. doi: 10.1186/s12859-016-1278-0, PMID: 27724866 PMC5057277

[ref22] Langmead B. Salzberg S. L. (2012). Fast gapped-read alignment with bowtie 2. Nat. Methods 9, 357–359. doi: 10.1038/nmeth.1923, PMID: 22388286 PMC3322381

[ref23] Liu X. Fan P. Che R. Li H. Yi L. Zhao N.. (2018). Fecal bacterial diversity of wild Sichuan snub-nosed monkeys (*Rhinopithecus roxellana*). Am. J. Primatol. 80:e22753. doi: 10.1002/ajp.22753 29635791

[ref24] Liu Z. X. Wang L. S. Chen M. (2021). Utilization and metabolism of intestinal microbial polysaccharides [J]. J. Microbiol. 61:13. doi: 10.13343/j.cnki.wsxb.20200478

[ref25] Lu J. Breitwieser F. P. Thielen P. Salzberg S. L. (2017). Bracken: estimating species abundance in metagenomics data. PeerJ Comput. Sci. 3:e104. doi: 10.7717/peerj-cs.104 PMC12016282 40271438

[ref26] Mandal S. Van T. W. White R. A. Eggesbo M. Knight R. Peddada S. D. (2015). Analysis of composition of microbiomes: a novel method for studying microbial composition. Microb. Ecol. Health Dis. 26:27663. doi: 10.3402/mehd.v26.27663, PMID: 26028277 PMC4450248

[ref27] Martin M. (2011). CUTADAPT removes adapter sequences from high-throughput sequencing reads. Bioinformatics 17, 10–12. doi: 10.14806/ej.17.1.200

[ref28] Mckenna A. Hanna M. Banks E. Sivachenko A. Cibulskis K. Kernytsky A.. (2010). The genome analysis toolkit: a MapReduce framework for analyzing next-generation DNA sequencing data. Genome Res. 20, 1297–1303. doi: 10.1101/gr.107524.110, PMID: 20644199 PMC2928508

[ref29] Muegge B. D. Kuczynski J. Knights D. Clemente J. C. Gonzalez A. Fontana L.. (2011). Diet drives convergence in gut microbiome functions across mammalian phylogeny and within humans. Science 332, 970–974. doi: 10.1126/science.1198719, PMID: 21596990 PMC3303602

[ref30] Nijboer J. Clauss M. (2006). The digestive physiology of Colobine Primates. Berlin: ResearchGate GmbH.

[ref31] Niu K. F. Liu W. Xiao Z. Wu A. K. Yang T. Y. Riondato I.. (2019). Exploring local perceptions of and attitudes toward endangered François’ langurs (*Trachypithecus francoisi*) in a human-modified habitat. Int. J. Primatol. 40, 331–355. doi: 10.1007/s10764-019-00091-0

[ref32] Parks B. W. Nam E. Org E. Kostem E. Norheim F. Hui S. T.. (2013). Genetic control of obesity and gut microbiota composition in response to high-fat, high-sucrose diet in mice. Cell Metab. 17, 141–152. doi: 10.1016/j.cmet.2012.12.007, PMID: 23312289 PMC3545283

[ref33] Schmieder R. Edwards R. (2011). Quality control and preprocessing of metagenomic datasets. Bioinformatics 27, 863–864. doi: 10.1093/bioinformatics/btr026, PMID: 21278185 PMC3051327

[ref34] Segata N. Izard J. Waldron L. Gevers D. Miropolsky L. Garrett W. S.. (2011). Metagenomic biomarker discovery and explanation. Genome Biol. 12:R60. doi: 10.1186/gb-2011-12-6-r60, PMID: 21702898 PMC3218848

[ref35] Spence C. Wells W. G. Smith C. J. (2006). Characterization of the primary starch utilization operon in the obligate anaerobe *Bacteroides fragilis*: regulation by carbon source and oxygen. J. Bacteriol. 188, 4663–4672. doi: 10.1128/JB.00125-06, PMID: 16788175 PMC1482989

[ref36] Su C. Zuo R. Liu W. Sun Y. Li Z. Jin X.. (2016). Fecal bacterial composition of Sichuan snub-nosed monkeys (*Rhinopithecus roxellana*). Int. J. Primatol. 37, 518–533. doi: 10.1007/s10764-016-9918-9

[ref37] Sun Y. F. Li Y. Zhang M. Zhang M. Li H. Y. Xu W. H.. (2017). Effects of adding tea seed meal to the diet on fat metabolism, immune function, and intestinal microbiota in broilers [J]. Feed Res. 3:6. doi: CNKI:SUN:SLYJ.0.2017-03-002

[ref38] Suzuki T. A. Ley R. E. (2020). The role of the microbiota in human genetic adaptation. Science 370:eaaz6827. doi: 10.1126/science.aaz6827 33273073

[ref39] Trosvik P. Rueness E. K. de Muinck E. J. Moges A. Mekonnen A. (2018). Ecological plasticity in the gastrointestinal microbiomes of ethiopian chlorocebus monkeys. Sci. Rep. 8:1. doi: 10.1038/s41598-017-18435-2, PMID: 29311667 PMC5758796

[ref40] Uhr G. T. Dohnalová L. Thaiss C. A. (2019). The dimension of time in host-microbiome interactions. Msystems 4, e00216–e00218. doi: 10.1128/msystems.00216-18 PMC6381226 30801030

[ref41] Versalovic J. Relman D. (2006). How bacterial communities expand functional repertoires. PLoS Biol. 4:e430. doi: 10.1371/journal.pbio.0040430, PMID: 17238278 PMC1750926

[ref42] Villar E. Farrant G. K. Follows M. Garczarek L. Speich S. Audic S.. (2015). Environmental characteristics of Agulhas rings affect interocean plankton transport. Science 348:1261447. doi: 10.1126/science.1261447 25999514

[ref02] West A. G. Waite D. W. Deines P. Bourne D. G. Digby A. McKenzie V. J.. (2019). The microbiome in threatened species conservation. Biol. Conserv. 229, 85–98. doi: 10.1016/j.biocon.2018.11.016

[ref43] Wood D. E. Salzberg S. L. (2014). Kraken: ultrafast metagenomic sequence classification using exact alignments. Genome Biol. 15:R46. doi: 10.1186/gb-2014-15-3-r46, PMID: 24580807 PMC4053813

[ref44] Wu G. D. Chen J. Hoffmann C. Bittinger K. Chen Y. Y. Keilbaugh S. A.. (2011). Linking long-term dietary patterns with gut microbial enterotypes. Science 334, 105–108. doi: 10.1126/science.1208344, PMID: 21885731 PMC3368382

[ref45] Zhu W. H. Lomsadze A. Borodovsky M. (2012). Ab initio gene identification in metagenomic sequences. Nucleic Acids Res. 38:e132. doi: 10.1093/nar/gkq275, PMID: 20403810 PMC2896542

