# Supplementary material for: The composition and function of the gut microbiota of Francois’ langurs (Trachypithecus francoisi) depend on the environment and diet
Source: Front Microbiol. 2023 Nov 16;14:1269492. doi: 10.3389/fmicb.2023.1269492 (PMC10687571; doi:10.3389/fmicb.2023.1269492)
Supplement: Supplementary file 1 [file Data_Sheet_1.docx]

**SUPPLEMENTARY MATERIALS**

Figure S1. 13 rarefaction curves approached a plateau, suggesting that the number of samples and sequencing depth were sufficient for experimental analyses

**
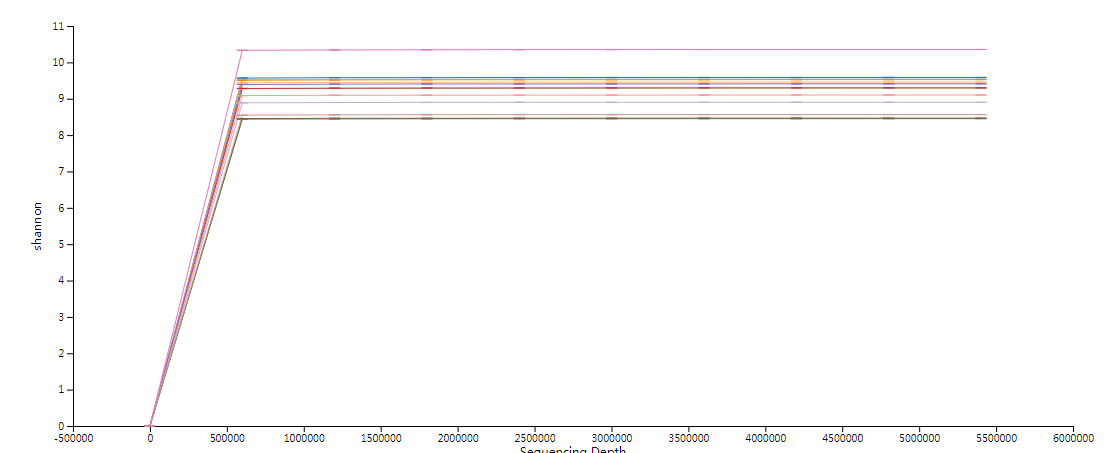

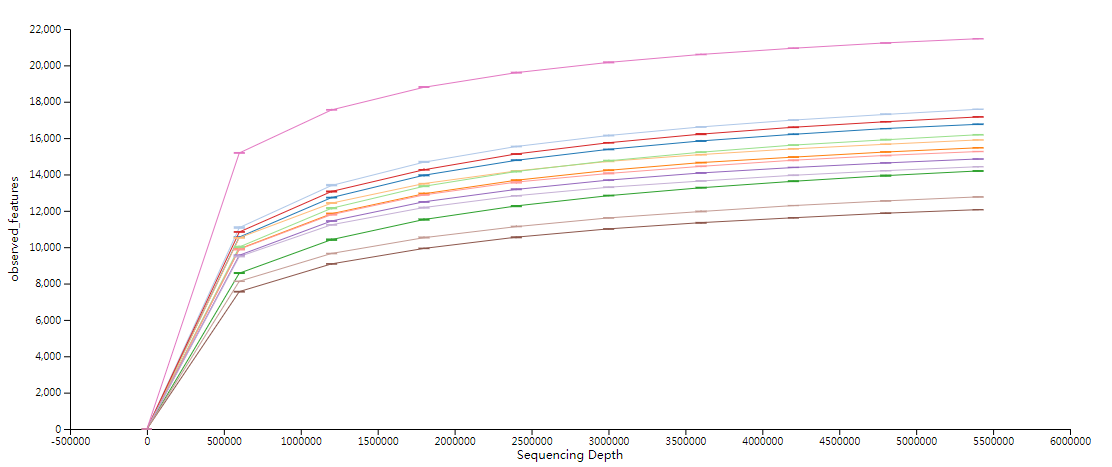
**

Figure S2. The relative abundance of KEGG pathways (level 2 function) for the microbial metagenome of the francois’ langurs under different environmental and dietary conditions.


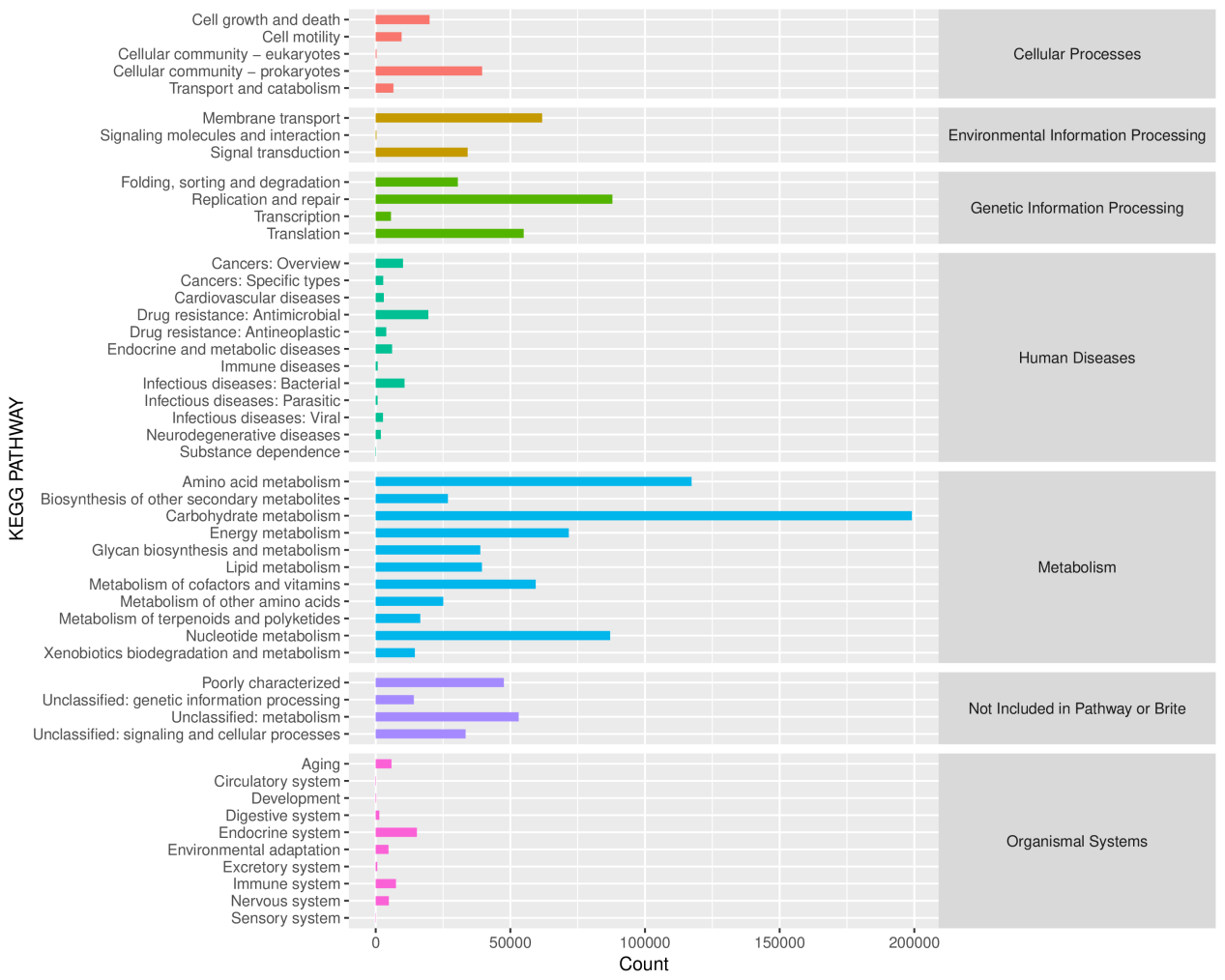


Table S1. Sequencing data statistics

| Sample  ID | Raw data（bp） | Vaild data  （bp） | Number of Reads | N (%) | GC (%) | Q20 (%) | Q30 (%) |
| --- | --- | --- | --- | --- | --- | --- | --- |
| AD1 | 57874868 | 57170478 | 8739105068 | 0.0012 | 47.07 | 98.08 | 94.31 |
| AD2 | 41344724 | 40718702 | 6243053324 | 0.0003 | 47.78 | 97.93 | 94.19 |
| AD3 | 48570036 | 47974764 | 7334075436 | 0.0012 | 47.47 | 98.06 | 94.27 |
| AD4 | 44852390 | 44313598 | 6772710890 | 0.0012 | 48.86 | 98.14 | 94.51 |
| W1 | 41214888 | 40639718 | 6223448088 | 0.0012 | 46.07 | 97.96 | 94.07 |
| W2 | 45980096 | 45457960 | 6942994496 | 0.0012 | 50.44 | 98.21 | 94.69 |
| W3 | 44181546 | 43650166 | 6671413446 | 0.0012 | 47.26 | 98.04 | 94.17 |
| W4 | 46061334 | 45417060 | 6955261434 | 0.0012 | 50.84 | 97.91 | 93.94 |
| W5 | 45927144 | 45184592 | 6934998744 | 0.0009 | 51.79 | 97.69 | 93.53 |
| C1 | 50792758 | 50169158 | 7669706458 | 0.0012 | 49.09 | 98.03 | 94.18 |
| C2 | 50745754 | 50121456 | 7662608854 | 0.0011 | 48.6 | 97.96 | 93.98 |
| C3 | 48383330 | 47818224 | 7305882830 | 0.0011 | 50.21 | 98.14 | 94.5 |
| C4 | 45752936 | 45228674 | 6908693336 | 0.0011 | 48.63 | 98.08 | 94.27 |

Table S2. The relative abundance of KEGG pathways (level 2 function) for the microbial metagenome of the francois’ langurs under different environmental and dietary conditions.

| Taxon | AD | W | C |
| --- | --- | --- | --- |
| Cellular Processes;Cell growth and death | 314.368875 | 307.76902 | 296.98505 |
| Cellular Processes;Cell motility | 288.472625 | 186.31048 | 115.918475 |
| Cellular Processes;Cellular community - eukaryotes | 1.185775 | 1.18686 | 0.077075 |
| Cellular Processes;Cellular community - prokaryotes | 195.6546 | 186.10338 | 183.434 |
| Cellular Processes;Transport and catabolism | 60.151275 | 66.7372 | 49.16735 |
| Environmental Information Processing;Membrane transport | 222.698525 | 240.78154 | 225.91365 |
| Environmental Information Processing;Signal transduction | 94.017925 | 91.14384 | 53.14405 |
| Environmental Information Processing;Signaling molecules and interaction | 0.0492 | 0.04036 | 0.191575 |
| Genetic Information Processing;Folding, sorting and degradation | 510.541875 | 492.01564 | 497.161625 |
| Genetic Information Processing;Replication and repair | 1313.17535 | 1157.8664 | 1284.325025 |
| Genetic Information Processing;Transcription | 104.75405 | 85.27744 | 86.07915 |
| Genetic Information Processing;Translation | 604.61355 | 565.35402 | 594.0761 |
| Human Diseases;Cancer: overview | 9.77625 | 12.14454 | 55.2723 |
| Human Diseases;Cancer: specific types | 0.946375 | 1.63686 | 0 |
| Human Diseases;Cardiovascular disease | 0.2449 | 0.31406 | 0.07865 |
| Human Diseases;Drug resistance: antimicrobial | 336.017775 | 333.46992 | 341.651075 |
| Human Diseases;Drug resistance: antineoplastic | 11.979375 | 9.44616 | 0.0447 |
| Human Diseases;Endocrine and metabolic disease | 20.823925 | 15.7961 | 37.5162 |
| Human Diseases;Immune disease | 0.915975 | 0.4858 | 0.2936 |
| Human Diseases;Infectious disease: bacterial | 136.439625 | 112.27556 | 86.94585 |
| Human Diseases;Infectious disease: parasitic | 10.964975 | 11.17566 | 9.801075 |
| Human Diseases;Infectious disease: viral | 6.46365 | 7.1462 | 1.406675 |
| Human Diseases;Neurodegenerative disease | 18.03025 | 16.28468 | 7.21125 |
| Human Diseases;Substance dependence | 0 | 0.03304 | 0 |
| Metabolism;Amino acid metabolism | 1572.76905 | 1522.13762 | 1451.978775 |
| Metabolism;Biosynthesis of other secondary metabolites | 383.6709 | 373.56134 | 378.56335 |
| Metabolism;Carbohydrate metabolism | 1743.455925 | 1738.57732 | 1666.211075 |
| Metabolism;Energy metabolism | 733.45085 | 712.50948 | 692.514025 |
| Metabolism;Glycan biosynthesis and metabolism | 704.714375 | 682.90222 | 704.248825 |
| Metabolism;Lipid metabolism | 541.29885 | 590.03144 | 554.998 |
| Metabolism;Metabolism of cofactors and vitamins | 1016.809325 | 1021.63918 | 1055.6498 |
| Metabolism;Metabolism of other amino acids | 932.2768 | 908.97964 | 982.440925 |
| Metabolism;Metabolism of terpenoids and polyketides | 235.056975 | 254.92258 | 244.863825 |
| Metabolism;Nucleotide metabolism | 298.215925 | 292.99044 | 323.7819 |
| Metabolism;Xenobiotics biodegradation and metabolism | 251.2351 | 265.39076 | 205.242375 |
| Organismal Systems;Aging | 28.69165 | 27.09108 | 23.052475 |
| Organismal Systems;Circulatory system | 0.737425 | 0.15424 | 0 |
| Organismal Systems;Development and regeneration | 4.067125 | 4.10322 | 0.4219 |
| Organismal Systems;Digestive system | 37.2582 | 24.71256 | 26.1692 |
| Organismal Systems;Endocrine system | 125.838475 | 97.54606 | 55.804725 |
| Organismal Systems;Environmental adaptation | 49.261275 | 37.79378 | 37.086375 |
| Organismal Systems;Immune system | 47.71795 | 28.96422 | 18.3269 |
| Organismal Systems;Nervous system | 17.852925 | 19.07176 | 5.546075 |
| Organismal Systems;Sensory system | 0.9433 | 1.0421 | 0 |
